# Reduced diversity and increased virulence-gene carriage in intestinal enterobacteria of coeliac children

**DOI:** 10.1186/1471-230X-8-50

**Published:** 2008-11-04

**Authors:** Ester Sánchez, Inmaculada Nadal, Ester Donat, Carmen Ribes-Koninckx, Miguel Calabuig, Yolanda Sanz

**Affiliations:** 1 Instituto de Agroquímica y Tecnología de Alimentos (CSIC), Apartado 73, 46100 Burjassot, Valencia, Spain; 2 Hospital Universitario La Fe, Avenida Campanar 21, 40009 Valencia, Spain; 3 Hospital General Universitario, Avenida Tres Cruces s/n 46014 Valencia, Spain

## Abstract

**Background:**

Coeliac disease is an immune-mediated enteropathology triggered by the ingestion of cereal gluten proteins. This disorder is associated with imbalances in the composition of the gut microbiota that could be involved in its pathogenesis. The aim of the present study was to determine whether intestinal *Enterobacteriaceae *populations of active and non-active coeliac patients and healthy children differ in diversity and virulence-gene carriage, so as to establish a possible link between the pathogenic potential of enterobacteria and the disease.

**Methods:**

*Enterobacteriaceae *clones were isolated on VRBD agar from faecal samples of 31 subjects (10 active coeliac patients, 10 symptom-free coeliac patients and 11 healthy controls) and identified at species level by the API 20E system. *Escherichia coli *clones were classified into four phylogenetic groups A, B1, B2 and D and the prevalence of eight virulence-associated genes (type-1 fimbriae [*fimA*], P fimbriae [*papC*], S fimbriae [*sfaD/E*], Dr haemagglutinin [*draA*], haemolysin [*hlyA*], capsule K1 [*neuB*], capsule K5 [*KfiC*] and aerobactin [*iutA*]) was determined by multiplex PCR.

**Results:**

A total of 155 *Enterobacteriaceae *clones were isolated. Non-*E. coli *clones were more commonly isolated in healthy children than in coeliac patients. The four phylogenetic *E. coli *groups were equally distributed in healthy children, while in both coeliac patients most commensal isolates belonged to group A. Within the virulent groups, B2 was the most prevalent in active coeliac disease children, while D was the most prevalent in non-active coeliac patients. *E coli *clones of the virulent phylogenetic groups (B2+D) from active and non-active coeliac patients carried a higher number of virulence genes than those from healthy individuals. Prevalence of P fimbriae (*papC*), capsule K5 (*sfaD/E*) and haemolysin (*hlyA*) genes was higher in *E. coli *isolated from active and non-active coeliac children than in those from control subjects.

**Conclusion:**

This study has demonstrated that virulence features of the enteric microbiota are linked to coeliac disease.

## Background

The human gastrointestinal tract is a complex ecosystem integrated by up to 10^14 ^bacteria. These microorganisms may belong to more than 500 different bacterial species, although 99% of the total community consists of only 30–40 species. The intestinal microbiota plays an important role in human health as they contribute to inhibiting pathogen colonization, boosting the immune system, and metabolising nutrients [1-3]. Alterations in the intestinal microbiota have also been linked to inflammatory bowel diseases (IBD), such as ulcerative colitis and Crohn's disease, as well as to other immune-related disorders. IBD patients have been shown to carry higher concentrations of mucosa-associated bacteria than control groups. In addition, the gut microbiota of these patients was characterized by a decrease in protective bacteria, such as *Bifidobacterium *and *Lactobacillus*, and an increase in harmful bacterial groups like *Bacteroides *and *Escherichia coli*. This phenomenon has been named "dysbiosis" [4,5].

*Escherichia coli *was the first bacterial specie to be identified in human gastrointestinal samples. This widespread commensal Gram-negative bacterium found in the intestinal tract of healthy individuals can occasionally cause intestinal and extraintestinal diseases. The pathogenic potential of *E. coli *stains could be influenced by host endogenous factors, as well as by the genetic structure and ecological distribution of the strains in a particular host. Human infections by *E. coli *strains commonly occur in immuno-compromised individuals or when the normal gastrointestinal barrier is broken [6], enabling opportunistic pathogens to evade host surveillance mechanisms. Phylogenetic studies based on the analysis of 38 metabolic enzymes, have shown that *E. coli *strains fall into four main phylogenetic groups, denominated "A", "B1", "B2" and "D" [7]. Strains causing extra-intestinal infections mainly belong to group B2, and to a lesser extent to group D, whereas commensal *E. coli *strains mostly belong to A and B1 groups [8]. *E. coli *strains belonging to B2 and D groups carry more virulence-associated genes than strains from A and B1 groups [9,10]. Therefore, the presence or absence of certain *E. coli *strains or an altered distribution of their relative abundance can enhance the pathogenic potential of the enteric population in predisposed individuals.

Coeliac disease is an immune-mediated enteropathology triggered by the ingestion of wheat-gluten and similar proteins of rye, barley and, probably, oats. Coeliac disease can be present at any age with a variety of clinical features; however, typical cases manifest in early childhood with gastrointestinal symptoms [11]. In coeliac patients, the inflammatory milieu caused by gluten antigens could lead to imbalances in gut microbial composition, characterised by higher numbers of Gram-negative bacteria and decreased numbers of beneficial Gram-positive bacteria, as compared to healthy individuals. In previous studies, *E. coli *proportions were significantly higher in duodenal biopsies of active coeliac patients than in controls [12]; moreover, a similar trend was detected in faeces although the differences were not so remarkable [13]. However, little is known about the possible association between enterobacterial population and coeliac disease pathogenesis.

The aim of the present study was to determine whether intestinal *Enterobacteriaceae *populations of active and non-active coeliac patients and healthy children differ in diversity and virulence-gene carriage, so as to establish a possible link between the pathogenic potential of enterobacteria and the disease.

## Methods

### Subjects and sampling

Thirty-one children were included in this study, 10 active coeliac patients, on a normal gluten-containing diet, showing clinical symptoms of the disease, positive coeliac serology markers and signs of severe enteropathy by duodenal biopsy examination (mean age 3.86 years, range 1.0–8.86 years); 10 symptom-free coeliac patients (non-active coeliac), who had been on a gluten-free diet for 1–2 years (mean age 6.2 years, range 1.0–12.0 years); and 11 healthy children without known food intolerance (mean age 3.51 years, range 0.1–7.75 years). None of the children included in the study had been treated with antibiotics for at least 1 month before the sampling time. Faecal samples were collected and immediately stored at 4°C, under anaerobic conditions (AnaeroGen, Oxoid, Hampshire, UK), and analysed in less than 12 hours. The study protocol was approved by the local Ethics Committee, and children were enrolled in the study after written informed consent was obtained from their parents.

### Isolation and identification of *Enterobacteriaceae *clones from faeces

Faecal samples (2 g wet weight) were 10-fold diluted in phospahate-buffered saline (PBS, 130 mM sodium chloride, 10 mM sodium phosphate, [pH 7.2]), and homogenized in a Lab Blender 400 Stomacher (Seward Medical, London, UK). Serial dilutions were made in PBS and aliquots were plated on Violet Red Bile Dextrose Agar, VRBD (Scharlau, Barcelona, Spain) and incubated under aerobic conditions at 37°C for 48 hours. Then, five individual *Enterobacteriaceae*-like colonies differing in size, shape, colour or mucoid appearance on VRBD agar were isolated and subcultured in Plate Count Agar (Scharlau, Barcelona, Spain) under aerobic conditions at 37°C for 24 hours. The identity of the presumptive *Enterobacteriaceae *isolates was confirmed by conventional microbiological methods, including colony and cellular morphology and Gram staining. The selected isolates were further identified at species level by using the API20E system (BioMerieux, Lyon, France), in order to select *E. coli *clones and non-*E. coli *clones.

### Phylogenetic classification and virulence-associated gene carriage of *E. coli *isolates

*Escherichia coli *isolates were assigned to one of the four phylogenetic groups A, B1, B2 or D by a triplex PCR technique as previously described [14]. The 25 μl PCR mixture consisted of 10 mM Tris-HCl (pH 8.3), 2.5 mM MgCl_2_, 1 μM of each primer pair for the genes *ChuA, YjaA *and *TspE4C2*, 200 μM, dNTPs and 2.5 U of Taq polymerase (Ecotaq, ECOGEN, Spain). A piece of a bacterial colony was collected directly from a plate and added to the reaction mixture as a source of DNA. The PCR programme was as follows: initial denaturalization at 94°C for 4 min; and 30 cycles of 5 s at 94°C, 10 s at 59°C; and a final extension step of 5 min at 72°C. The PCR products were separated in a 2% agarose gel electrophoresis and visualised by ethidium-bromide staining. The isolates were assigned to phylogenetic groups as follows: ChuA^+ ^YjaA^+^, group B2; ChuA^+^YjaA^-^, group D; ChuA^- ^TspE4C2^+ ^group B1; ChuA^- ^TspE4C2^- ^group A.

The same isolates were also characterized by two sets of multiplex PCR as described by Nowrouzian et al. (2001) [15]. The first PCR detected the presence of the following virulence-associated genes: *fimA *(type-1 fimbriae), *papC *(P fimbriae), *sfaD/E *(S fimbriae) and *draA *(Dr haemagglutinin). The second PCR detected the presence of the following genes: *hlyA *(haemolysin), *neuB *(capsule K1), *KfiC *(capsule K5) and *iutA *(aerobactin). The 25 μl PCR reaction mixture consisted of 10 mM Tris HCl (pH 8.3), 1.5 mM of MgCl_2 _for the first multiplex PCR and 2.0 mM MgCl_2 _for the second multiplex PCR, 0.45 μM of each primer, 100 μM dNTPs and 2.5 U of Taq polymerase (Ecotaq, ECOGEN, Spain). A piece of a bacterial colony was used as a source of DNA as indicated above.

#### Statistical analyses

Differences in prevalence of different species, phylogenetic groups or virulence genes were established by applying the chi-square test, and when appropriate by the two-tailed Fisher's exact test and Kolmogorov-Smirnof test. In each case, analyses were carried out with the Statgraphics software (Manugistics, Rockville, MD), and statistical differences were established at *P *value below 0.05.

## Results

### Diversity of Enterobacteriaceae and *E. coli *clones from coeliac and healthy children

Of the 31 individuals analyzed in this study, only 14 (45%) carried *Enterobacteriaceae *species other than *E. coli*. The latter species was always recovered in healthy individuals and in eight of them (73%) at least one other non-*E. coli *species was detected. Non-*E. coli *species were detected in only four (40%) active and two non-active coeliac patients (20%). Non-*E. coli *clones were recovered more abundantly in healthy children than in non-active coeliac patients (*P *= 0.03).

*Citrobacter freundii, Enterobacter agglomerans, Klebsiella oxytoca *and *Escherichia vulneris *were identified in healthy children harbouring non-*E. coli *species. *Klyuvera *spp, *Enterobacter cloacae, Citrobacter freundii, Shigella *spp, *Chryseomonas luteola *and *Klebsiella oxytoca *were also isolated in active coeliac disease children and *Klebsiella oxytoca, Aeromonas hydrophila/caviae *and *Klebsiella pneumoniae *were identified in non-active coeliac patients.

### Phylogenetic classification of *E. coli *clones from coeliac and healthy children

**Figure 1:**
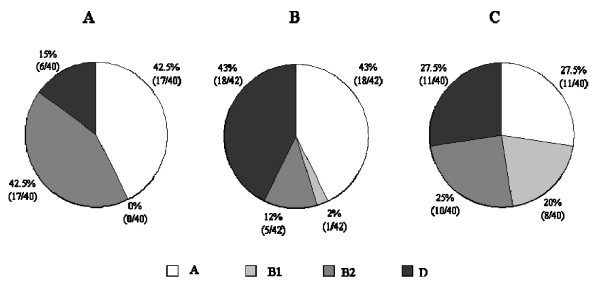
**Distribution of the phylogenetic *E. coli *groups in active coeliac patients (A), non-active coeliacs (B) and healthy children (C). **A and B1 commensal groups and B2 and D virulent groups. Data are expressed as percentage (number of positive clones/number of total analysed clones).

The phylogenetic classification of *E. coli *clones isolated from faeces of the three groups of children is shown in Figure 1. The total number of isolates belonging to both commensal groups (A+B1) and the total number of isolates belonging to both virulent groups (B2+D) did not differ significantly between the three populations of children under study. However, differences were observed in the abundance of each of the four phylogenetic groups. In healthy children, A and B1 *E. coli *groups were equally distributed, while in active and non-active coeliac patients almost all the commensal isolates belonged to group A (*P *< 0.001), revealing lower *E. coli *diversity in clones from coeliac patients. Significant differences were found between the prevalence of virulent clones isolated from active or non-active coeliac patients and healthy children. In healthy individuals, virulent isolates were equally represented by both groups B2 and D. In contrast, group B2 was the main virulent group isolated from active coeliac patients (*P *= 0.0002), and group D was that most common in non-active coeliac patients (*P *= 0.0001).

### Virulence-associated gene carriage in *E. coli *clones from coeliac and healthy children

The prevalence of virulence-associated genes in commensal groups (A+B1) and virulent groups (B2+D) of *E. coli *clones isolated from active and non-active coeliac and control children are shown in Table 1. Overall, virulent *E. coli *clones (B2 + D) from healthy individuals carried fewer virulence-associated genes than those from active and non active coeliac patients. Both active and non-active coeliac patients had a significantly higher prevalence of P fimbriae gene (*papC*), (*P *= 0.002–0.050) than healthy controls. In addition, type-1 fimbriae gene (*fimA*) prevalence was higher in both active and non-active coeliac patients than in healthy controls but the differences were not significant (*P *= 0.06). Capsule K1 gene (*neuB*) was also more prevalent in both active and non-active coeliac patients than in healthy controls; however, differences were not significant. Capsule K5 gene (*KfiC*) was significantly more common in virulent *E. coli *isolates from active coeliac children than in those from non-active coeliac children (*P *= 0.02), suggesting its association with the active phase of the disease. Haemolysin gene (*hlyA*) prevalence was higher in both coeliac disease subjects than in healthy controls, showing significant differences between non-active coeliac disease patients and controls (*P *= 0.01). In commensal groups (A+B1), S fimbriae (*sfaD/E*) gene prevalence was significantly higher in non-active coeliac patients than in healthy and active coeliac patients (*P *= 0.04 and *P *= 0.02), whereas capsule K1 gene (*neuB*) was significantly less common in commensal *E. coli *isolates from active coeliac children than in the other groups (*P *< 0.001). Moreover, aerobactin gene (*iutA*) was more common in commensal *E. coli *clones isolated from healthy controls than those from active coeliac individuals (*P *< 0.001).

**Table 1:** Prevalence of virulence-associated genes in A+B1 (commensal) and B2+D (virulent) *E. coli *clones isolated from active and non-active coeliac disease patients and healthy children

Virulence factor (gene)	Virulent B2 + D clones (%)*			Commensal A + B1 clones (%)*		
	
	Active coeliac (n = 23)	Non-active coeliac (n = 23)	Healthy (n = 21)	Active coeliac (n = 17)	Non-active coeliac (n = 19)	Healthy (n = 19)
Type 1 fimbriae (*fim A*)	18 (78)^a^	18 (78)^a^	10 (48)^a^	11 (65)^a^	14 (82)^a^	16 (84)^a^
S fimbriae (*sfaD/E*)	8 (35)^a^	9 (39)^a^	8 (38)^a^	0^a^	6 (35) ^b^	1 (5) ^a^
P fimbriae (*papC*)	5 (22)^a^	9 (39)^a^	0^b^	0^a^	0^a^	4 (21)^a^
Dr haemagglutinin (*draA*)	0^a^	0^a^	0^a^	0^a^	0^a^	0^a^
Haemolysin (*hlyA*)	5 (22)^ab^	9 (39)^b^	1 (5)^a^	0^a^	1 (6)^a^	4 (21)^a^
K1 (*neuB*)	8 (35)^a^	5 (22)^a^	5 (24)^a^	0^a^	10 (59) ^b^	8 (42)^b^
K5 (*KfiC*)	6 (26)^a^	0^b^	2 (10)^ab^	0^a^	0^a^	1 (5)^a^
Aerobactin (*iutA*)	4 (17)^a^	4 (17)^a^	7 (33)^a^	1 (6)^a^	6 (35)^ab^	12 (63) ^b^

*Different letters (a to b) in the same row and within the same phylogenetic group (virulent B2+D or commensal A+B1) indicate significant differences in the gene-carriage.

**Figure 2:**
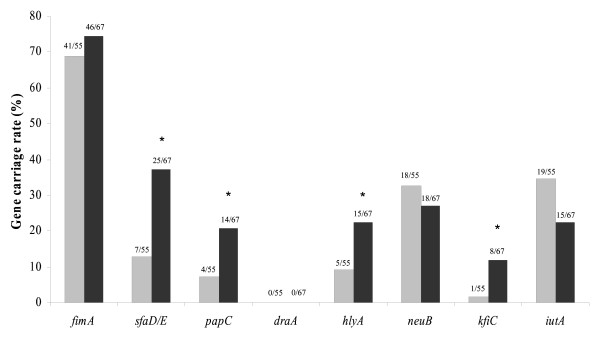
**Prevalence percentage of virulence-associated genes in A+B1 commensal (grey bars) and B2+D virulent (black bars) *E. coli *groups.** Data are expressed as percentage (number of positive clones/number of total analysed clones). *Significant difference at *P *< 0.05 by applying the Fisher's exact test.

Figure 2 shows the prevalence of virulence-associated genes in commensal groups (A+B1) and virulent groups (B2+D) of *E. coli *clones isolated in this study. S fimbriae (*sfaD/E*), P fimbriae (*papC*), haemolysin (*hlyA*) and capsule K5 (*KfiC*) genes were significantly more common in virulent *E. coli *clones than in commensal clones (P < 0.05). Accordingly, P fimbriae (*papC*), haemolysin (*hlyA*) and capsule K5 (*KfiC*) genes prevalence was associated with one or both coeliac patient groups, which would suggest that *E. coli *clones from patients have a higher pathogenic potential than those from controls. No differences were detected in the carriage of type 1 fimbriae (*fimA*), Dr haemagglutinin (*draA*), capsule K1 (*neuB*) and aerobactin (*iutA*) genes between the commensal and virulent *E. coli *groups. Furthermore, the distribution of these genes was either less remarkable or bore no relationship with coeliac disease.

## Discussion

Significant differences were revealed in the diversity and virulence-gene carriage of *Enterobacteriaceae *and *E. coli *clones isolated from faeces of active and non-active coeliac patients and healthy controls, suggesting that this bacterial group could be primarily or secondarily involved in coeliac disease.

*E. coli *was the most common member of the *Enterobacteriaceae *family isolated from human faecal samples of healthy and coeliac disease children. This result is consistent with a previous study of 49 coeliac and healthy children, in which the main *Enterobacteriaceae *species detected was *E. coli*. In the aforementioned study, the ratio of *E. coli *to *Enterobacteriaceae *was greater in active coeliac children than in healthy controls [13]. Furthermore, biopsy specimens taken from paediatric patients with inflammatory bowel disease also revealed that *E. coli *was the most common *Enterobacteriaceae *species detected in MacConkey agar [16]. Thus, a reduction in the relative abundance of non-*E. coli *species is associated with coeliac disease and could favour the predominance of harmful clones in the gut ecosystem.

Although several *Enterobacteriaceae *colonies were often selected from a sample, because they differed morphology and were suspected to represent different strains, they often turned out to be identical regarding their phylogenetic classification and virulence-associated gene patterns, usually some of them represented the same clone. This can be explained by the fact that only one clone usually dominates human gastrointestinal gut so that the other clones are not detectable [17].

The phylogenetic groups A, B1, B2 and D were found in different proportions in healthy, active coeliac and non-active coeliac patients. This fact also suggests links between lower diversity of intestinal *E. coli *population and coeliac disease. In healthy children, isolates of each of the four groups were found in equal proportions. By contrast, isolates belonging to groups A and B2, followed by those of group D dominated the composition of *E. coli *microbiota in active coeliac children, while integrants of group B1 were not isolated; group D was the most common in non-active coeliac disease, followed by groups A, B2, and B1. These results reflect imbalances in the composition of the four *E. coli *phylogenetic groups in coeliac disease children, revealing the most remarkable differences between non-active coeliac patients and healthy controls. In patients with IBD, such as Crohn's disease and ulcerative colitis, B2 and D phylogenetic groups were more prevalent than in the control group. Moreover, a relationship was established between the presence of serine protease autotransporter proteins (SPATE) or adhesins in B2+D-positive *E. coli *isolates [18]. In active coeliac patients, the dominance of group B2 virulent clones could provide pathogenic inflammatory factors either causative or consequence of the inflammatory status associated with gluten intake in the active phase of this disorder creating a vicious circle. Nowrouzian et al. [19] concluded that group D strains preferentially colonize infants with less complex intestinal microbiota, since this group is more abundant than others in one-year-old infants. Both genetic make-up and gluten-free diet could contribute to changes in the gut milieu of non-active coeliac patients, leading to a predominant colonization of the virulent D group in detriment to the others. In fact, lower total bacterial populations have been detected in duodenal samples taken from this population group when compared with active coeliac patients and controls [12].

Overall, *E coli *clones of the virulent phylogenetic groups (B2+D) isolated from active and non-active coeliac patients, carried a higher number virulent genes than those from healthy controls. A higher prevalence of P fimbriae gene (*papC*) was found in coeliac patients as compared to healthy children, regardless of the phase of this disorder; however, the association of type-1 fimbriae (*fimA*) and S fimbriae (*sfaD/E*) gene carriage with coeliac disease was not so evident. Significant differences in the carriage of type-1 fimbriae (*fimA*) gene were not detected in the group of children under study but *E. coli *clones from active and non-active coeliac disease isolates tended to carry fewer type-1 fimbriae (*fimA*) genes than those from healthy children. Previous studies have indicated that the proportion of type-1 fimbriated *E. coli *is lower in IgA-deficient subjects than in control individuals [20,21] and this may also be the case for coeliac disease patients since this disease is associated with IgA deficiency. *E. coli *adhesins, including P fimbriae, confer mannose-resistant (MR) adherence to intestinal epithelial cells. MR adhesions are well-know virulence factors in urinary-tract infection, septicaemia and meningitis. Strains that persist in the human intestinal microbiota (resident strains) are more often P fimbriated, whereas S fimbriated *E. coli *are not associated with long-term persistence in the gut of healthy individuals [20,22]. Thus, the significantly higher prevalence of P fimbriae *E. coli *clones in treated and untreated coeliac patients constitutes a novel link between gut microbiota and coeliac disease, and revealed that gut health may be compromised in these subjects, even when subjected to a gluten-free diet.

A higher prevalence of capsular K5 gene (*kfiC*) of *E. coli *clones belonging to the virulent groups was associated with active coeliac disease, while a similar but less remarkable trend was found in non-active coeliac patients, as compared to controls. Capsular polysaccharides are known to render bacterial surfaces hydrophilic and negatively charged, making the bacterium resistant to entrapment in mucus. In addition, capsules contribute to virulence by protecting bacteria from phagocytosis and possibly from serum killing, in part by blocking activation of the alternative complement pathway [23,15].

Moreover, a higher prevalence of the haemolysin (*hlyA*) gene in *E coli *clones of the virulent groups was associated with non-active coeliac patients in particular. α-Haemolysin is the most common cytolytic protein secreted by haemolytic *E. coli *strains. Haemolysin activity might contribute to persistence by attacking enterocytes and releasing nutrients for the bacteria. In fact, *E. coli *appears to use membrane lipids as its main nutrient source in the large intestine [23,24].

It has also been confirmed that *E. coli *clones of the virulent groups (B2 + D) carried more virulent-associated genes than those of commensal groups (A + B1), with the exception of the aerobactin gene (*iutA*). Remarkably, genes for S (*sfaD/E*) and P fimbriae (*papC*), K5 capsule (*kfiC*) and haemolysin (*hlyA*) were significantly more common in virulent *E. coli *clones than in commensal strains of our population groups. In agreement, genes coding for virulence-associated genes have been found more frequently in pathogenic strains than in commensal strains [8,23]. In addition, virulent *E. coli *isolates (B2 + D) have previously been shown to carry virulence-associated genes more commonly than commensal clones [10,25,26]. Virulence-associated genes are usually encoded on pathogenicity islands (PAIs) providing a mechanism for coordinated horizontal transfer of virulence genes, thus favouring dissemination of pathogenic determinants, as could be the case in the gut ecosystem of coeliac patients [27].

## Conclusion

This study has demonstrated changes in *Enterobacteriaceae *diversity and increases in virulence-gene carriage in *E. coli *clones isolated from coeliac patients when compared to those from healthy controls. Thus, the results support the hypothesis that dysbiosis may constitute a virulence factor contributing to pathogenesis and full expression of coeliac disease. Further studies should be carried out to correlate the virulence gene carriage with specific pathogenic roles that *E. coli *clones could play in this disorder.

## Competing interests

The authors declare that they have no competing interests.

## Authors' contributions

ES and IN carried out the microbiological, molecular and statistical analyses. ED, CR and MC collected the samples and clinical data. YS conceived and coordinate the study. YS and ES wrote the manuscript. All authors read, reviewed and approved the final version of the manuscript.

## Pre-publication history

The pre-publication history for this paper can be accessed here:


